# A Cross-Sectional Survey to Estimate the Cat Population and Ownership Profiles in a Semirural Area of Central Italy

**DOI:** 10.1155/2016/3796872

**Published:** 2016-08-17

**Authors:** Andrea Carvelli, Francesca Iacoponi, Paola Scaramozzino

**Affiliations:** Istituto Zooprofilattico Sperimentale del Lazio e della Toscana “M. Aleandri”, Via Appia Nuova 1411, 00178 Roma, Italy

## Abstract

Understanding animal population size and its demographic features is essential to address Public Health policies as well as to provide valuable information to pet industries and veterinary practitioners. Nevertheless, official data on feline population are not available worldwide. In the present study, the owned cat population size, its demographic attributes, and the ownership profiles have been investigated through a face-to-face questionnaire in a semirural area of Central Italy. The human : cat ratio was equal to 6.8 (95% CI: 5.7–7.5); 29.3% of households own at least one cat. The majority of cats were living in a rural area (67.8%) and outdoors, were neutered (70.5%), and were fed with commercial food (54.8%) and they visited a veterinarian 1-2 times a year (43.3%). The cat ownership was strongly associated with people living in a rural area and owning another pet. As the cat owned population was mainly kept outdoors in rural areas, the possible relation between the owned and the stray animals is worthy to be monitored in future researches. Our study revealed that the feline owned population was larger than expected and that social and economic human factors do not influence the cat ownership. Health Authorities and veterinary practitioners should promote responsible ownership to increase the veterinary care, to intensify the official identification, and to properly manage the outdoor lifestyle.

## 1. Introduction 

The understanding of animal population data in terms of abundance, spatial distribution, and demographic structure is pivotal for health and economic purposes: animal diseases control, zoonoses risk assessment, animal welfare issues, stray population management, and valuable information for veterinary industries and practitioners [1, 2]. An accurate estimate of the size and features of an animal population is therefore essential to address health policies properly. At present, official feline population data are not available worldwide since the cat census is not always recognised as an important tool for Public Health. However, since 2012 in France, Greece, Estonia, and Slovakia cat identification is required by law. In Iceland, Sweden, Belgium, Portugal, and Bulgaria a Feline Registry is in the process of being set up or is being discussed [3]. Furthermore, during a workshop carried out in 2013 on pets welfare organized by the European Veterinarian Association, Presidency of European Union (EU) Council, and other associations, pet identification and registration were identified as necessary and should be made mandatory in all EU countries [4].

So far, the only available data are provided by industry or* ad hoc* surveys [5]. A number of papers have been published on owned cat population estimate. Some studies measured the human : cat ratio and found it to be between 5.2 and 9.9 [1, 6, 7] while other ones estimated the percentage of cat-owning household (HH) and found it to be between 15.0% and 31.1% [1, 6–13].

In 2012, the European Pet Food Industries estimated that 72 million European households owned at least a dog or a cat. Russia had the highest number of cats with an estimate of 18 million cats, followed by France (11 million), Germany, and the United Kingdom (8 million) and Italy where 7,482,000 cats lived [14]. A very similar estimate, 7,400,000 cats, was assessed by another source [15].

In Italy, cat identification and registration are on a voluntary basis. A microchipped cat can be registered in the Feline Unit of the public regional Canine Registry or in a private Feline Registry managed by the Italian Association of Veterinarians and supported by a pharmaceutical company. At present, in the whole country, 239,825 cats are recorded in the public registry (mainly implemented by the feral cat colonies managed by Local Health Unit) and 26,687 cats in the private one [16, 17].

Indeed, these data represent a clear underestimation of the real owned population and cannot be considered absolutely informative.

Furthermore, information regarding the demographics of cat populations and the ownership profiles is underprovided. Factors as age, neutering, housing, diet, frequency of veterinary visits, source of cats, and their trend represent important issues for epidemiological studies and they have been examined in the present study. A few papers investigated human factor influencing the cat ownership, such as gender, age, presence of children, presence of another pet, house location, respondent educational level, and the owning of a cat [10, 11].

**Figure 1:**
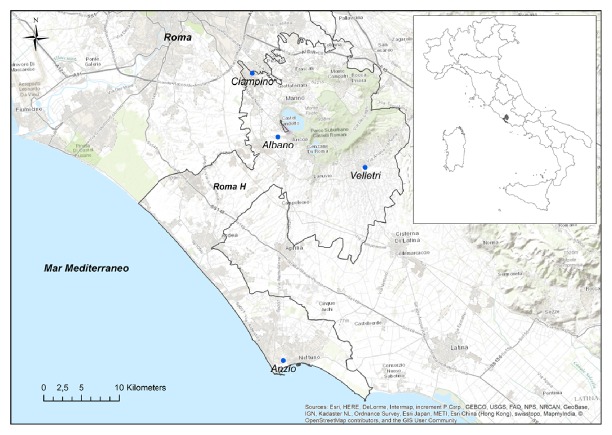
The study area (dark grey): Health District “Roma H” and Health Care Centres where interviews were performed (black dots).

The researchers of the Istituto Zooprofilattico Sperimentale del Lazio e della Toscana carried out a cross-sectional survey on pet ownership through a face-to-face questionnaire. The present study was performed in the Health District “Roma H” (Figure 1) which is located southeast of Rome (Central Italy).

The first aim of this study was to estimate the owned feline population size and its demographic structure. The secondary aim was to identify the human factors influencing the cat ownership.

## 2. Materials and Methods

### 2.1. Study Area and Survey

The study area covers the Health District “Roma H” which is part of Rome Province (13.6% of the territory and 11.1% of the population). It covers 726.7 km^2^ with a population of 539,445 inhabitants (population density: 742.3 per km^2^) distributed in 209,566 households [18]. It is located between the Tyrrhenian coastal area and inland territories and includes 21 municipalities surrounded by farmed lands and wooded areas. The average elevation is 232 meters above sea level and the average temperature is 14.7°C. 62.4% of the land is used for agricultural activities, 14.3% is covered by forest, 7.0% is represented by urban area, and 16.3% is classified as another use [19]. As the study area includes both rural and urban areas, it can be considered representative either of a countryside where several towns are present or of a peripheral territory of a metropolitan area.

The survey was carried out during weekdays between the months of July and December 2013 in the waiting rooms of 4 Health Care Centres of the National Health System (Albano Laziale, Anzio, Ciampino, and Velletri). Systematic sampling was performed by selecting one from every four persons amongst the general diagnostic patients. Survey participation was requested within the framework of a research project on human and animal tumours funded by the Ministry of Health.

The definition of cat ownership used in this survey was based on the respondent's definition. The interviewers underlined the care provided by the owner in terms of regular feeding and health status. The physical restriction of the animal was not taken into consideration.

The requested sample size was 668 questionnaires, assuming a 2.5% standard error and 95% confidence level. The expected prevalence of people owning cats was 12% considering an estimated feline population of 7,400,000 cats [15] and a human population of 60,782,668 in Italy [18].

### 2.2. Questionnaire Design

A structured questionnaire with 16 questions was designed by veterinarians, epidemiologists, and social scientists and administered by 7 trained interviewers. Each person selected for the interview was firstly asked whether they were resident of the study area and provided information on the survey. The gender and the age group of those that chose not to participate in the survey were recorded for completeness. All questions were closed-ended. The participants were asked about whether they owned a cat and/or other pets, the number of owned pets, the characteristics of the owned cat (sex, neutering, breed, and age), the source of the cat (born in house, found, gift, adopted from shelter, or purchased), the kind of environment the cat usually lived in (urban/rural area and mainly outdoor/indoor space), the kind of feeding (homemade, commercial, or mixed), and the annual average veterinary care frequency (never, 1-2 times a year, or 3 or more times). Furthermore, household size, the presence of children, and the registration to the public Feline Registry were investigated. The final section of the questionnaire covered the personal information of the participant (gender, age, marital status, education level, occupation, and living place: an urban/rural area).

### 2.3. Data Analysis

The information gathered during the survey was entered into a Microsoft Access® (Microsoft Office® 2003) database developed* ad hoc* for this study. Data were reported as absolute frequencies and percentages, as mean and standard deviation (SD), and as median and minimum and maximum values (min/max).

The association between urban/rural environment and the other categorical variables was evaluated by Chi-square or Fisher's exact test. Normality of the distributions was tested by Shapiro-Wilks test and the difference between age distributions was calculated by Mann-Whitney* U* test. Univariable logistic regression was performed considering veterinary care frequency (recoded as a dichotomous value: never/1 or more times a year) as outcome variable and other factors as independent variables. The significant independent variables were included in stepwise multivariable logistic regression.

Univariable and multivariable logistic analyses were also performed considering cat owning as a dependent variable and other factors as independent variables. A *p* value < 0.05 (two-tailed) was considered statistically significant.

In order to avoid overestimating cat owners, the number of interviewed people was adjusted considering the total number of the family members. Hence, the estimated number of cats was calculated using the following formula:

(1) Estimated number of cats=Roma  H Population·∑icats∑ifamily members,

where *i* is the interviewed subject. The binomial 95% confidence interval (95% CI) was calculated for the estimated cat population. All statistical analyses were performed by StataSE® v.12.0 (StataCorp, Texas, USA).

## 3. Results

Out of a total of 668 people selected in the 4 Health Care Centres, 519 (77.7%) agreed to participate in the survey and 484 resided in the study area. Only the latter were considered in the analyses. The total number of interviewed people and their family members was 1242. The number of interviewed subjects can be considered as the number of households. In the present study, the mean number of persons per HH was 2.6, similar to the Italian mean, 2.4 [18].

### 3.1. Feline Population Estimate

Overall, 342 (70.7%) interviewed subjects did not have cats while 142 (29.3%) people owned at least one. 60 interviewed subjects (42.3%) owned only one cat, 41 (28.9%) two cats, 23 (16.2%) three cats, 11 (7.7%) four cats, and 7 (4.9%) five or more, counting up to 184. The mean number of cats per HH was 0.4 while it was 1.3 per cat-owning HH.

The cat population size estimate based on formula (1) was 79,918 (95% CI: 71,731–94,175).

The human : cat ratio was 6.8 (95% CI: 5.7–7.5).

### 3.2. Demography of Cat Population

**Figure 2:**
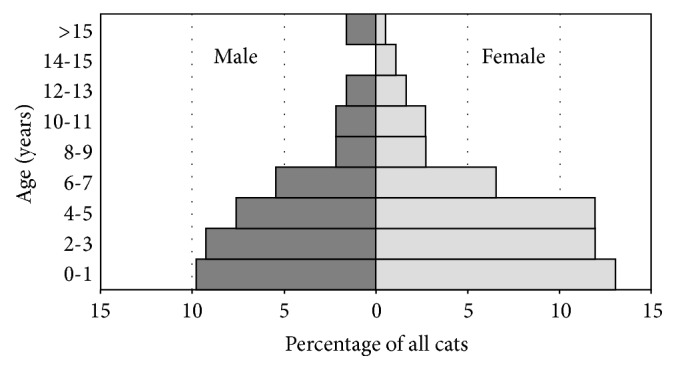
Feline population pyramid.

43.2% of cats were male with a mean age of 4.8 years (SD 4.7), whereas 56.8% were female with a mean age of 4.3 years (SD 3.8). The age distribution reported in the population pyramid (Figure 2) shows a similar shape for both sexes; 67.1% and 70.8% of males and females were younger than 5 years old, respectively.

The majority of the cats were European Shorthair breed or crossbreed (95.6%), neutered (70.5%), fed with commercial food (54.8%), visited by a veterinarian 1-2 times a year (43.3%), found (48.5%), not registered in the Feline Unit of the public Canine Registry (94.4%), and living in a rural area (67.8%) and mainly outdoors (81.7%) (Table 1).

Neutering was performed in 70.5% of the animals, with the, with the percentage being significantly higher in females (76.7% versus 55.2%) than in males.

A significant association (*p* < 0.01) between cat neutering and gender was observed: 76.7% of females were sterilized whereas 55.2% of males were neutered.

The urban/rural area was considered to explore the potential associations between categories of cats and the environment where they lived. The frequencies reported in Table 2 showed a significant association between cats' age, the average veterinary care, indoor or outdoor living, HH size, and the living environment. The majority of cats that lived in rural area were young, did not visit a veterinarian annually, lived mainly outdoors, and lived with 3 or more people. The cat population living in an urban area had a homogeneous age group and HH size distribution and were submitted to veterinary care more frequently (Table 2).

In Table 3, the average veterinary care was considered as the outcome variable, after grouping and recoding as a dichotomous value. The cats usually visited by a veterinarian (1 or more times a year) were often found or received as a gift (83.2%), neutered (85.7%), and fed with commercial food (61.9%) and were more likely to live in an urban area (41.0%).

### 3.3. Ownership Profiles

In our survey, 60.6% of the cat owners also had other pets: 58.5% had a dog and 2.1% had birds (parrots, chickens), hamsters, geese, rabbits, fishes, or turtles. The characteristics of the interviewed people owning or not a cat are reported in Table 4. The interviewed subject's gender was significant (OR = 1.93, *p* < 0.01), with women being the 71.6% of the cat owners. Owning a cat was weakly associated among age classes, 40–59 versus 0–39 (*p* = 0.048), and among separated versus single status classes (*p* = 0.037), while cat owners mainly lived in rural areas and were likely to own other pets as confirmed by the multivariable analysis (*p* < 0.001).

## 4. Discussion

### 4.1. Feline Population Estimate

The estimation of animal population size and demographic structure is a key factor for animal health governance to perform epidemiological studies, to implement surveillance plans, to control stray animals, and to educate pet owners on zoonosis. The most recent papers estimating pet population use the percentage of households owning cats instead of human : cat ratio. This method is likely used because surveys are carried out by telephone, reaching HH and not counting its members. In the present study, we preferred to estimate the cat population using the human : cat ratio, since we believe that this data format is more readily comparable in areas with a different people/household ratio.

The human : cat ratio of 6.8 (95% CI: 5.7–7.5) identified in the present survey was lower (i.e., larger cat population) if compared to the previous studies in other Western countries [1, 6, 9, 13, 20]. Considering the percentage of HH where at least one cat lives, the prevalence found in the present study, 29.3%, was much higher than results reported in previous studies carried out in Italy and Ireland (prevalence between 10.4% and 19.0%) [9, 10, 13, 20] and similar to what found was in UK [11] and Australia [8] (26.0% and 25.8–31.1%, resp.).

The mean of cat per owning-cat HH was 1.3, while in all the other cited studies it was around 1.7. These findings clearly indicate a spread population, few cats distributed over many HH.

Regardless of both approaches (human : cat ratio and HH prevalence), this study suggests the presence of an abundant feline population in the study area, higher than what was expected considering national survey and published papers [9, 13, 15]. This can be partially explained by the rural environment, considering that many people owned cats that lived preferably outdoors, although they were entirely cared for in terms of feeding, neutering, and veterinary care. However, our estimate of the human : cat ratio is not so different from the values published in previous studies, suggesting that the ratio between cat and human population can be considered rather constant in Western countries.

### 4.2. Survey Design

Many surveys are carried out by reaching people through mail service, landline telephone, or door-to-door. It is believed that nowadays a survey carried out via landlines (or based on a selection by telephone registry), which are becoming more and more obsolete, would not be reliable, because of a too high selection bias. Then, we consider the face-to-face questionnaire as the method to be preferred, though costly and time consuming, with a consequent risk of the population overestimation, because this method is likely the most accurate one in reducing the selection bias [5].

The questionnaires were applied in Health Care Centres to general people attending for routine diagnostic exams (blood analyses, general medical visits, and clinical examinations). In Italy, the Health Care System provides comprehensive coverage to all citizens [21, 22] and no private insurance system is involved. Considering that routine diagnostic exams are performed in the Health Care Centres and homogenously among age, sex, and socioeconomic levels, the sampled population can be considered representative of the general population.

The questionnaire was completely anonymous and this reduced the measurement bias. Nonresponse bias, that is, non-pet owners avoiding the interview, was reduced focusing the attention on general medical topic and avoiding the mention of pets.

### 4.3. Demography of Cat Population

The population pyramid shows that female and young age classes were more represented. The higher number of females compared to males cats, in all the cohorts of births, can be partially explained by higher natural mortality of males living outdoors for traumas and infectious diseases spread caused by fighting in breeding or territorial behaviour [2]. Another hypothesis is a possible preference of people to own a female rather than a male, but more causes should be investigated. However, this finding needs to be taken into consideration when managing sex-related issues such as planning neutering campaigns or managing diseases. Moreover, the population pyramid is skewed toward young age classes, as expected in a high fertility species [23], which suggests that the feline population size is likely to remain stable or increase in the future, unless a significant change in the factors affecting population growth will happen.

Among the papers that examine cat demographics, only a few reported the percentage of neutering and this value varies considerably: 45.0% in Central Italy [13], 76.6% in Ireland [10], and 97.3% in Sidney [2]. In the present survey neutering was performed in 70.5% of the animals, with the percentage being significantly higher in females than in males. Although this study identified a low percentage of animals left sexually intact amongst owned cats, compared with 57% of a previous report in a close province [13], uncontrolled breeding remains a strong possibility when considering that the majority of cats in this study lived mainly outdoors and were therefore in contact with stray cats that were probably not neutered. Moreover, in the group living mainly outdoors, neutering was less performed and the females were more neutered than males, but entire males play an important role in the uncontrolled growth of animal population due to the high prevalence of mating in a large territory.

The presence of a contact between the owned and the stray population can be supposed because the main part of the owned cats lived in rural areas and outdoors. In addition, the survey highlighted that the source of 44% of owned cats was found, a finding that is consistent with what happens in a close province [13]. The consequences of this relation for the owned population in terms of spread of infectious diseases, fighting, and uncontrolled breeding are worthy of future investigation [11]. Information campaigns should be oriented to raise awareness in the public opinion on this problem.

The high percentage of cats receiving a low level veterinary care, 32.5% of cats not attending a veterinary clinic once a year, has a certainly negative impact on infectious diseases spread and on the cats' welfare.

Despite recent accomplishments in the creation of the Feline Registry, a very small number of cats were microchipped and registered. A huge effort has to be made by the appointed Health Bodies to achieve a good percentage of feline enrolment.

Association analysis and logistic regression were performed on the variables we considered most important, environment where the cat was living and veterinary care frequency, respectively. In animals living in the rural area, the mean age was significantly lower. This younger population was also less likely visited by a veterinarian and lived mainly outdoors. These factors could result in higher exposure to mortality causes such as fighting, infectious diseases, and traumas. More frequent veterinary visits were related to owning a found cat versus a born-in-house cat, a higher percentage of neutering, feeding the animal with a commercial product, and living in an urban area. Owners should be encouraged to visit a veterinary clinic more frequently, in order to get the appropriate vaccination program for their pets and to neuter them at an early stage, especially the cats roaming free in rural areas.

### 4.4. Ownership Profiles

The univariable analysis shows that the profile which is more likely to own a cat corresponded to female, age class 40–59, and separated (marital status). Downes et al. and Murray et al. [10, 11] also reported more female respondents owning cat, but when this finding is balanced by the sex distribution in the household members, it loses significance. The age and the marital status had a weak association and in other studies these factors were inversely related to the pet ownership. At the multivariable level, a strong association was confirmed only for people living in a rural area. Owning a cat appears to be more practical in a rural area (which probably means a detached house or at least the presence of a garden), but this aspect should be more appropriate for dogs, because cats can comfortably live indoors. These findings may also reflect the will to own any pet rather than a cat, probably for an increasing need of human-animal bond, related to the health benefits associated with pet ownership.

Interestingly, many factors that could affect or have been found to affect in other studies the cat ownership, household size, presence of children, and occupation, did not have a significant effect in the present survey. However only few papers on cat owners' profiles have been published [10, 11, 13] and more researches are worthy of publishing. Our findings suggest that there are no reliable predictors for the cat ownership.

## 5. Conclusion

The up-to-date information on pet population size and its demographic features is crucial in Public Health. Our study suggests that the feline owned population can be more abundant than expected and that social and economic human factors do not influence the ownership of a cat. The demographic trend and the relation between the owned and the stray animals are worthy to be monitored in future researches. Health Authorities and veterinary practitioners should promote responsible ownership to increase the veterinary care, to intensify the official identification, and to properly manage the outdoor lifestyle.

## Figures and Tables

**Table 1:** Demographic characteristics of 184 cats.

		*N*	%	*Missing*
Sex	Male	73	43.2	*15*
	Female	96	56.8	

Neutered	Yes	103	70.5	*38*
	No	43	29.5	

Breed	European Shorthair/crossbreed	174	95.6	*2*
	Pedigree	8	4.4	

Source	Born in house	40	24.0	*17*
	Found	81	48.5	
	Gift	5	3.0	
	Adopted	1	0.5	
	Purchased	40	24.0	

Living environment	Urban area	58	32.2	*4*
	Rural area	122	67.8	
	Indoors	21	18.3	*69*
	Outdoors	94	81.7	

Feeding	Homemade	9	5.0	*5*
	Commercial	98	54.8	
	Mixed	72	40.2	

Veterinarian	Never	58	32.5	*6*
	1-2 times	77	43.3	
	3 or more times	43	24.2	

Feline Registry	Yes	2	1.1	*5*
	No	169	94.4	
	Do not know	8	4.5	

**Table 2:** Descriptive and association analysis for cats that lived in urban or rural area.

Where the cats live: *N* = 184 (missing = 4)			
	Urban area (*N* = 58)	Rural area (*N* = 122)	*p* value^*∗*^
*Sex*			
Male	28 (48.3)	43 (40.2)	0.316
Female	30 (51.7)	64 (59.8)	
* Missing*	*0*	*15*	
*Age in years*			
Median (min/max)	4.5 (0.1/23)	3.0 (1/21)	0.768
0–3	24 (41.4)	70 (57.4)	0.044
4–6	17 (29.3)	34 (27.9)	
>6	17 (29.3)	18 (14.8)	
*Missing*	*0*	*0*	
*Breed*			
European/crossbreed	54 (93.1)	116 (96.7)	0.441
Pedigree	4 (6.9)	4 (3.3)	
*Missing*	*0*	*2*	
*Source*			
Born in house	12 (24.5)	28 (24.4)	0.999
Found/gift	35 (71.4)	83 (72.2)	
Adopted/purchased	2 (4.1)	4 (3.4)	
*Missing*	*9*	*7*	
*Neutered*			
Yes	38 (77.5)	62 (65.9)	0.151
No	11 (22.5)	32 (34.1)	
*Missing*	*9*	*28*	
*Feeding*			
Homemade	2 (3.6)	6 (4.9)	0.327
Commercial	26 (47.3)	70 (57.9)	
Mixed	27 (49.1)	45 (37.2)	
*Missing*	*3*	*1*	
*Veterinarian*			
Never	9 (15.8)	48 (41.0)	<0.01
1-2 times per year	34 (59.7)	41 (35.0)	
3 or more times per year	14 (24.5)	28 (24.0)	
*Missing*	*1*	*5*	
*Where do they live?*			
Indoors	19 (47.5)	2 (2.7)	<0.001
Outdoors	21 (52.5)	73 (97.3)	
*Missing*	*18*	*47*	
*Household size*°	*N* = 54	*N* = 84	
1 person	4 (8.0)	5 (6.0)	<0.01
2 persons	22 (44.0)	16 (19.3)	
3 or more persons	24 (48.0)	62 (74.7)	
*Missing*	*4*	*1*	
*Children*°			
Yes	12 (25.0)	27 (32.1)	0.387
No	36 (75.0)	57 (67.9)	
*Missing*	*6*	*0*	

^*∗*^Chi-square or Fisher's exact test for categorical variables and Mann-Whitney *U* test for continuous variables.

°Data from 142 owners of cats (4 missing).

**Table 3:** Descriptive, univariable, and multivariable logistic analysis for cats that visited a veterinarian.

Veterinarian: *N* = 184 (missing = 6)						
	Never *N* = 58	1 or more times a year *N* = 120	Univariable logistic model		Multivariable logistic model	
			OR (95% CI)	*p* value	OR (95% CI)	*p* value
*Sex*						
Male	26 (44.8)	46 (43.8)	1.0 (0.5–1.9)	0.900		
Female	32 (55.2)	59 (56.2)				
* Missing*	*0*	*0*				
*Age (years)*						
0–3	29 (50.0)	64 (53.4)	—	—		
4–6	19 (32.8)	31 (25.8)	0.7 (0.4–1.5)	0.411		
>6	10 (17.2)	25 (20.8)	1.1 (0.5–2.7)	0.775		
*Missing*	*0*	*0*				
*Breed*						
European/crossbreed	57 (98.3)	112 (94.1)	3.6 (0.4–29.4)	0.240		
Pedigree	1 (1.7)	7 (5.9)				
* Missing*	*0*	*1*				
*Source*						
Born in house	27 (49.1)	13(12.1)	—		—	
Found/gift	28 (50.9)	89 (83.2)	**6.6 (3.0–14.4)**	**<0.001**	**3.62 (1.1–13.0)**	**<0.05**
Adopted/purchased	0 (0)	5 (4.7)	—		—	
* Missing*	*3*	*13*				
*Neutering*						
Yes	14 (32.6)	84 (85.7)	**12.4 (5.3–29.2)**	**<0.001**	**10.5 (3.7–30.1)**	**<0.01**
No	29 (67.4)	14 (14.3)				
* Missing*	*15*	*22*				
*Feeding*						
Homemade	7 (12.3)	2 (1.7)	—		—	
Commercial	21 (36.8)	73 (61.9)	**12.2 (2.4–63.0)**	**<0.01**	**13.0 (1.2–146.4)**	**<0.05**
Mixed	29 (50.9)	43 (36.4)	**5.2 (1.1–6.8)**	**<0.05**	**21.7 (1.8–259.8)**	**<0.05**
* Missing*	*1*	*2*				
*Where the cat lives*						
Urban area	9 (15.8)	48 (41.0)	—		—	
Rural area	48 (84.2)	69 (59.0)	**3.7 (1.7–8.3)**	**<0.001**	**4.0 (1.2–13.5)**	**<0.05**
* Missing*	*1*	*3*				
*Household size*°	*N* = 43	*N* = 93				
1 person	1 (2.4)	6 (6.7)	—	—		
2 persons	11 (26.8)	26 (28.9)	0.4 (0.1–3.5)	0.398		
3 or more persons	29 (70.8)	58 (64.4)	0.4 (0.1–3.1)	0.346		
* Missing*	*2*	*3*				
*Children*°						
Yes	15 (35.7)	25 (28.4)	0.6 (0.3–1.1)	0.089		
No	27 (64.3)	63 (71.6)				
* Missing*	*1*	*5*				

°Data from 142 owners of cats (6 missing).

**Table 4:** Descriptive, univariable, and multivariable logistic analysis of survey participants demographic characteristics.

Owning of cats						
	No (*N* = 342)	Yes (*N* = 142)	Univariable logistic model		Multivariable logistic model	
			OR (95% CI)	*p* value	OR (95% CI)	*p* value
*Gender*						
Male	145 (43.3)	38 (28.4)	**1.9 (1.3–3.0)**	**<0.01**	1.2 (0.6–2.4)	0.094
Female	190 (56.7)	96 (71.6)				
*Missing*	*7*	*8*				
*Age (years)*						
0–39	91 (26.9)	26 (18.3)	—		—	—
40–59	156 (45.2)	75 (52.8)	**1.7 (1.1–2.8)**	**<0.05**	0.7 (0.3–2.4)	0.218
≥60	91 (26.9)	41 (28.9)	1.6 (0.9–2.8)	0.118	0.5 (0.3–1.9)	0.681
*Missing*	*4*	*0*				
*Marital status*						
Single	56 (17.7)	18 (12.9)	—	—	—	—
Married	222 (70.0)	94 (67.1)	1.3 (0.7–2.4)	0.354	0.7 (0.3–1.7)	0.923
Separated	22 (6.9)	17 (12.1)	**2.4 (1.1–5.5)**	**<0.05**	4.7 (0.3–17.1)	0.277
Widowed	17 (5.4)	11 (7.9)	2.0 (0.8–5.1)	0.139	2.3 (0.6–11.5)	0.263
*Missing*	*25*	*2*				
*Education level*						
Elementary school	29 (9.3)	15 (11.2)	—	—		
Middle school	94 (30.9)	44 (32.8)	0.9 (0.4–1.9)	0.785		
High school	154 (49.6)	51 (38.1)	0.6 (0.3–1.3)	0.211		
University	34 (10.9)	24 (17.9)	0.8 (0.6–3.1)	0.454		
*Missing*	*31*	*8*				
*Occupation*						
Home working	159 (49.8)	64 (46.0)	—	—		
Office	99 (31.0)	44 (31.7)	1.1 (0.7–1.7)	0.672		
Other	61 (19.2)	31 (22.3)	1.3 (0.8–2.2)	0.380		
*Missing*	*23*	*3*				
*Where the respondent lives*						
Urban area	117 (59.7)	54 (38.3)	**2.8 (1.6–5.1)**	**<0.001**	**3.8 (1.8–7.8)**	**<0.001**
Rural area	79 (40.3)	87 (61.7)				
*Missing*	*146*	*1*				
*Household size *						
1 person	11 (5.4)	9 (6.6)	—	—		
2 persons	63 (30.7)	38 (27.7)	0.7 (0.3–1.9)	0.537		
3 or more persons	131 (63.9)	90 (65.7)	0.8 (0.3–2.1)	0.710		
*Missing*	*137*	*5*				
*Children*						
Yes	68 (32.7)	41 (30.1)	0.9 (0.6–1.4)	0.620		
No	140 (67.3)	95 (69.9)				
*Missing*	*134*	*6*				
*Other pets*						
Yes	157 (45.9)	86 (60.6)	**1.8 (1.2–2.7)**	**<0.001**	**4.3 (2.2–8.5)**	**<0.001**
No	185 (54.1)	56 (39.4)				
*Missing*	*0*	*0*				
